# Dietary fatty acid intake in childhood and the risk of islet autoimmunity and type 1 diabetes: the DIPP birth cohort study

**DOI:** 10.1007/s00394-022-03035-2

**Published:** 2022-10-25

**Authors:** Leena Hakola, Anna-Leena Vuorinen, Hanna-Mari Takkinen, Sari Niinistö, Suvi Ahonen, Jenna Rautanen, Essi J. Peltonen, Jaakko Nevalainen, Jorma Ilonen, Jorma Toppari, Riitta Veijola, Mikael Knip, Suvi M. Virtanen

**Affiliations:** 1 Unit of Health Sciences, Faculty of Social Sciences, Tampere University, Tampere, Finland; 2 Research, Development and Innovation Center, Tampere University Hospital, Tampere, Finland; 3 VTT Technical Research Centre of Finland, Tampere, Finland; 4 Health and Well-Being Promotion Unit, Finnish Institute for Health and Welfare, Helsinki, Finland; 5 Immunogenetics Laboratory, Institute of Biomedicine, University of Turku, Turku, Finland; 6 Institute of Biomedicine, Research Centre for Integrative Physiology and Pharmacology, and Centre for Population Health Research, University of Turku, Turku, Finland; 7 Department of Pediatrics, Turku University Hospital, Turku, Finland; 8 PEDEGO Research Unit, Department of Pediatrics, Medical Research Center, University of Oulu, Oulu, Finland; 9 Department of Children and Adolescents, Oulu University Hospital, Oulu, Finland; 10 Pediatric Research Center, Children’s Hospital, University of Helsinki and Helsinki University Hospital, Helsinki, Finland; 11 Research Programs Unit, Diabetes and Obesity, University of Helsinki, Helsinki, Finland; 12 Department of Paediatrics, Tampere University Hospital, Tampere, Finland; 13 Center for Child Health Research, Tampere University and Tampere University Hospital, Tampere, Finland

**Keywords:** Fatty acids, Children Dietary intake, Diabetes mellitus, Type 1, Autoimmune diseases, Cohort study

## Abstract

**Purpose:**

The aim was to study the associations between dietary intake of fatty acids in childhood and the risk of islet autoimmunity and type 1 diabetes (T1D).

**Methods:**

The prospective Finnish Type 1 Diabetes Prediction and Prevention (DIPP) Study included children with genetic susceptibility to T1D born between 1996 and 2004. Participants were followed up every 3 to 12 months up to 6 years for diet, islet autoantibodies, and T1D. Dietary intake of several fatty acids at the age of 3 months to 6 years was assessed 1–8 times per participant with a 3-day food record. Joint models adjusted for energy intake, sex, HLA genotype and familial diabetes were used to investigate the associations of longitudinal intake of fatty acids and the development of islet autoimmunity and T1D.

**Results:**

During the 6-year follow-up, 247 (4.4%) children of 5626 developed islet autoimmunity and 94 (1.7%) children of 5674 developed T1D. Higher intake of monounsaturated fatty acids (HR 0.63; 95% CI 0.47, 0.82), arachidonic acid (0.69; 0.50, 0.94), total *n*-3 fatty acids (0.64; 0.48, 0.84), and long-chain *n*-3 fatty acids (0.14; 0.04, 0.43), was associated with a decreased risk of islet autoimmunity with and without energy adjustment. Higher intake of total fat (0.73; 0.53, 0.98), and saturated fatty acids (0.55; 0.33, 0.90) was associated with a decreased risk of T1D only when energy adjusted.

**Conclusion:**

Intake of several fatty acids was associated with a decreased risk of islet autoimmunity or T1D among high-risk children. Our findings support the idea that dietary factors, including *n*-3 fatty acids, may play a role in the disease process of T1D.

**Supplementary Information:**

The online version contains supplementary material available at 10.1007/s00394-022-03035-2.

## Introduction

Type 1 diabetes (T1D) is a chronic, immune-mediated disease that is preceded by the appearance of circulating islet autoantibodies (islet autoimmunity) before clinical diagnosis [1]. Children with certain human leukocyte antigen (HLA) genotypes are at increased risk of T1D, however, environmental factors are believed to explain the changes in the disease incidence during past decades [1–3]. Several environmental candidates, including several dietary factors may contribute to the risk of T1D [2, 4].

Dietary fatty acids and their metabolites may have the potential either to accelerate or to inhibit the processes leading to T1D by affecting inflammation, immunity, gut microbiota, gut permeability, and gene expression, for example [5–8]. Mechanistic studies suggest that saturated fatty acids (SFAs) promote pro-inflammatory and, monounsaturated fatty acids (MUFAs) and polyunsaturated fatty acids (PUFAs) promote anti-inflammatory states; however, findings from dietary interventions in human are partly inconsistent [9–11].

The associations between dietary fatty acid intake in children and the risk of islet autoimmunity or progression to T1D has so far been reported only in the Diabetes Autoimmunity Study in the Young (DAISY). In DAISY, *n*-3 and *n*-6 PUFAs were studied, and higher dietary intake of *n-*3 PUFAs in childhood was associated with a decreased risk of islet autoimmunity [12] but nor progression to T1D [13]. To our understanding, total fat, SFA, and MUFA intake in relation to T1D related outcomes has not been studied before. In addition to dietary fatty acid intake, consumption of cod liver oil, rich in long-chain *n*-3 PUFAs, during the first year of life was associated with lower risk of T1D in a Norwegian case–control study [14]. Studies including fatty acid biomarkers that to some extent reflect dietary intake but mostly metabolism, suggest that higher levels of *n*-3 fatty acids may be associated with a decreased risk of islet autoimmunity, although the findings are not fully consistent [12, 15–17]. In biomarker studies higher proportion of some SFAs, MUFA, and conjugated linoleic acids have been associated with an increased risk of islet autoimmunity with some inconsistency [16–18]. Recently, some linoleic acid and alpha-linolenic acid-related oxylipins were reported to be associated with a decreased risk of T1D in the DAISY study [19].

Our aim was to study the associations between children’s dietary intake of total fat and individual fatty acids and the risk of developing islet autoimmunity and T1D. This study is the largest to report such associations so far and will add to the current knowledge by utilizing longitudinally and prospectively collected food record data that yields daily intakes of several fatty acids, and by linking those to regularly collected islet autoantibody and T1D follow-up data. We hypothesized that higher intake of *n*-3 PUFAs during childhood decreases and higher intake of SFAs increases the risk of islet autoimmunity and T1D.

## Subjects and methods

### Study design and population

The Finnish Type 1 Diabetes Prediction and Prevention (DIPP) Study is a large population*-*based birth cohort study of children with HLA*–*conferred susceptibility to T1D [20]. Children carrying the genotypes HLA-DQB1*0⁄03:02 and DQB1*03:02 ⁄ *x* (*x* indicates alleles other than DQB1*03:01 or DQB1*0602/3 until March 1997 and other than DQB1*02 or DQB1*06:02 thereafter) were eligible for the follow-up in the Tampere and Oulu University hospitals until September 2004. In the nutrition study within the DIPP Study, 54 350 children born in the Tampere and Oulu University hospitals between September 1996 and September 2004 were screened for HLA from cord blood sample, 8293 (15.2%) were eligible, 7782 (14.3%) were invited, and 6080 children (78% of those invited) participated in the islet autoantibody follow-up (Online resource, Fig. 1). The children were invited to follow-up visits at study clinics at intervals of 3 to 12 months up to age 6 years for food consumption and up to age 15 years for islet autoantibodies and T1D. In this analysis, the follow-up was limited to 6 years. The inclusion criteria for this study included at least one autoantibody assessment, and at least one completed food record day with information on energy intake at or before the autoantibody assessment (islet autoimmunity cohort) and at least one completed food record day with information on energy intake (T1D cohort). Children repeatedly positive to at least one islet autoantibody with at least one food record day at or after first seropositivity were included in the progression analyses (progression cohort) (Online resource, Fig. 1). Written informed consent was obtained from the parents for genetic testing of their newborn infant from the cord blood sample and another one for participation in the follow-up. The study adheres to the Declaration of Helsinki, and the ethics committees of Oulu and Tampere University Hospitals approved the study protocol.

### Dietary assessment

Children’s diet was assessed with 3-day food records (including 1 weekend and 2 weekdays) at 3, 6, and 12 months and 2-, 3-, 4-, and 6-years of age, and for part of the children at 5-years. The collection of food consumption data has been described previously [21]. The procedures included the training of the study nurses and research nutritionists, written instructions to families to write down all foods and drinks (with portion sizes, recipes including dietary fats, preparation methods, and brand names) the child has consumed, as well as checking and completing the food records at return and probing for missing items [21]. Families were asked to specify the type of dietary fats and milks used in recipes and cooking.

Trained nutritionists entered the food record data. Food and nutrient calculations are based on constantly updated well-maintained national food composition database Fineli, Finnish Institute for Health and Welfare, Finland, that includes over 8000 food items. Calculated dietary components include energy (kilocalories), total fats, SFA, myristic acid, palmitic acid, MUFA, *n*-6 PUFA, linoleic acid, arachidonic acid, conjugated linoleic acid, *n*-3 PUFA, alpha-linolenic acid, and long-chain *n*-3 PUFA. Total energy intake was calculated based on food records. For those who were breastfed at the age of 3, 6, or 12 months, we estimated the total energy intake based on age, body weight, and the expected energy deposition needed for growth [22]. Amount of breastmilk was calculated based on the difference between expected energy expenditure and energy intake reported in food records among those who were breastfed [23]. Fat and fatty acid intakes presented are based on foods and drinks (including breast milk) and dietary supplements. Fatty acid values in the database are based on chemical analysis of food samples, recipe calculations and adopting values from other sources, e.g., other databases, scientific literature, and food labeling. Fineli recipe calculations are performed according to EuroFIR guidelines [24] and nutrient retention factors are based on report of National Food Administration, Sweden [25]. Total fat values are based on chemical analyses or when not, conversion factors are used to convert total fatty acids to fat [26]. During the years 2012–2016, we reviewed and updated the values of fatty acids for the most important fatty acid sources in the Fineli database by screening of possible errors, correcting observed errors and by thorough quality checking of the new database versions. This process improved the accuracy of the fatty acid data.

### Islet autoimmunity, T1D, and progression to T1D

Children were screened for islet cell antibodies (ICA) at 3 to 12-month intervals as described before [27]. When a child seroconverted to positivity for ICA for the first time, all preceding and subsequent samples from that participant were analyzed for insulin autoantibodies (IAA), glutamic acid decarboxylase antibodies (GADA) and islet antigen-2 antibodies (IA-2A). ICA were quantified by a standard indirect immunofluorescence method, IAA, GADA, and IA-2A with specific radiobinding assays. Islet autoimmunity was defined as repeated positivity for ICA and at least one biochemical autoantibody (IAA, GADA, IA-2A) by February 2017 or having T1D. The date of diagnosis of T1D was obtained in May 2017 from Finnish Pediatric Diabetes Register [28, 29] which covers approximately 92% of children diagnosed with T1D by the age of 15 years in Finland. T1D diagnosis was based on WHO Criteria [30]. In the present study, children not found in the register were considered T1D-free. In progression analysis, risk of T1D was assessed among children who were repeatedly positive to at least one autoantibody.

### Genetic methods

HLA-DQ was genotyped using panels of sequence-specific oligonucleotide probes, as described before [31]. Genotypes HLA-DQB1*02/03:02 represent “high” and HLA-DQB1*03:02/x (*x* ≠ *02, *03:01, *06:02) “moderate” risk for T1D.

#### Background characteristics

Information on familial diabetes (any type) in first-degree relative (yes, no), and maternal vocational education (none, vocational, secondary vocational, university studies or degree) was collected with a questionnaire after delivery. Duration of any breastfeeding was the age when the child received breast milk for the last time based on questionnaires checked at each early study visit (3, 6, 12, 18, and 24 months).

### Statistical methods

Joint models that combine longitudinal and survival data into a single model [32], were used to investigate the association between the dietary fatty acid intake and the development of islet autoimmunity and T1D. Joint models allowed modeling the longitudinal intake of the fatty acid and simultaneously investigate its association with the time until the development of islet autoimmunity or T1D.

In joint models, the fatty acid intake from 3 months up to 6 years was modeled using piecewise natural cubic splines with three knots in the linear mixed effects (LME) submodels. The locations of knots were specified through an algorithm that selected best suitable selection of knots by fitting all relevant combinations and selecting the best fitting model based on the Bayesian information criteria. The algorithm required that there had to be at least two records before the first knot, and after the last knot, and 1 year between the knots. The survival submodel was built using the structure of the Cox proportional hazards regression model. The children were followed until the date of the development of islet autoimmunity/T1D, or the age of 6 years. The baseline hazard of the joint model was set as a piecewise constant with change points at the ages of 1.99 and 3.99 given the follow-up of 6 years. In progression analyses the time from first seroconversion until T1D or the age of 6 years was used as time scale. The fatty acid intakes measured during that time were used in LME submodels. In addition, if measurement of fatty acid intake prior to first seroconversion was available for an individual, linear interpolant between the measurement prior to and the measurement following first seroconversion was fitted, and the intake at first seroconversion was approximated from the interpolation line. This was done to ensure the appropriate fit for the individual LME curves at the beginning of the follow-up period (before the first available measurement) since the ages, and thus the intakes, at first seroconversion (start of follow-up) varied a lot between individuals, and no one had intake measured exactly at first seroconversion. The baseline hazard of the joint model for progression was set as a piecewise constant with change points at the times of 1.99 and 3.99 years given the follow-up from first seroconversion until T1D or the age of 6 years. The models were otherwise similar to those assessing T1D in whole cohort.

**Table 1:** Distribution of all participating children by background variables and outcomes

	Islet autoimmunity cohort		Type 1 diabetes cohort		Progression cohort	
	Total *N* = 5626 No. (%)	Islet autoimmunity *N* = 247 No. (%)	Total *N* = 5674 No. (%)	Type 1 diabetes *N* = 94 No. (%)	Total *N* = 505 No. (%)	Type 1 diabetes *N* = 64 No. (%)
Sex						
Male	2988 (53.1)	148 (59.9)	3010 (53.0)	54 (57.4)	285 (56.4)	42 (65.5)
Female	2638 (46.9)	99 (40.1)	2664 (47.0)	40 (42.6)	220 (43.6)	22 (34.4)
HLA-conferred risk^a^						
High	1102 (19.6)	77 (31.2)	1109 (19.5)	35 (37.2)	116 (23.0)	23 (35.9)
Moderate	4524 (80.4)	170 (68.8)	4565 (80.5)	59 (62.8)	389 (77.0)	41 (64.1)
Familial diabetes^b^						
Yes	333 (5.9)	30 (12.2)	333 (5.9)	14 (14.9)	43 (8.5)	9 (14.1)
No	5080 (90.3)	211 (85.4)	5125 (90.3)	78 (83.0)	452 (89.5)	54 (84.4)
Missing information	213 (3.8)	6 (2.4)	216 (3.8)	2 (2.1)	10 (2.0)	1 (1.6)

^a^High-riskgenotypes: HLA-DQB1(*02/*03:02) and Moderate-risk genotypes: HLA-DQB1(*03:02/ x);  x  ≠ *02, *03:01,*06:02)

^b^Includes all types of diabetes

The joint models were estimated within a Bayesian framework using Markov chain Monte Carlo (MCMC) algorithms. In Bayesian estimation, inference is based on the distribution over the parameter space (full posterior distribution) instead of maximum likelihood. The parameter values from the submodels of the joint model were given as prior information for the full posterior distribution. Three chains were set for the MCMC, leading to three distributions of the possible values for each model parameter. The Gelman–Rubin diagnostic [33] was used to check the similarity of the obtained distributions for the parameters, and thus assess the convergence of the MCMC sampler. The diagnostic values less than 1.1 were considered as convergence. In addition, density plots and traceplots were visually inspected.

The joint models were run separately for each fatty acid, and for the sum variables without and with energy-adjustment for three outcomes. All models were adjusted for sex (male or female), HLA (high or moderate risk), and familial diabetes of any type (yes or no), as these variables have been previously found to be potential confounders. Progression analyses were also adjusted for age at seroconversion. Energy-adjustment was done using multivariate nutrient density method [34]: fatty acid intake (grams or 0.1 g) was divided by the total energy intake (in megajoules), and the received variable was included in a model as a covariate together with the total energy intake. The models provided the posterior mean estimates as hazard ratios (HR) and 95% credible intervals (CI). The Bayesian CIs represent the interval in which the population parameter lies with a given probability. A current value association structure was used, and thus the HR at a given point in time t is provided for a one-unit (1 g, 100 mg, 10 mg g/MJ, 100 mg/MJ, or 10 mg/MJ) increase in the longitudinal value of the fatty acid intake at the same time point t.

We performed a sensitivity analyses to assess, whether reverse causality could explain the association between energy intake and risk of T1D [35] and the protective associations of energy-adjusted fat and SFA intake. This is because T1D can increase eating and drinking before the diagnosis. We did this by excluding the food records from 18 months before the T1D diagnoses from those who developed T1D in the T1D cohort. 18 months was selected due to the observed changes in insulin metabolism already 18 months before diagnosis [36].

The advantage of using the joint modeling framework was that these models can accommodate imbalanced data. Therefore, we were able to use all food records available for each child; children with more frequently reported dietary data contributed more to the analysis. In addition, each child was allowed to have her/his own intake trajectory through the inclusion of subject-specific spline coefficients (the random effects) in the LME model.

Previous studies suggest that fatty acids may play a role in the development of islet autoimmunity especially in infancy [15]. Therefore, we used Cox proportional hazards regression model with the mean energy-adjusted fatty acid intake at ages 3 and 6 months as a time-independent covariate and adjusted the analyses for sex, HLA genotype, and familial diabetes, to study the risk of developing islet autoimmunity and T1D during 6-year follow-up. Maternal education was tested in the models due to its association with offspring’s early fatty acid intake but not included in the final model as inclusion did not change the results. Furthermore, to understand whether breastfeeding modifies the association between early fatty acid intake and risk of islet autoimmunity and T1D, we added the interaction term between “being breastfed at 3 months (yes/no) and “mean fatty acid intake at ages 3 to 6 months” in Cox regression model in addition to the previously listed covariates.

Multiple testing was controlled for using the false discovery rate (FDR) method (a step-up procedure using a 0.05 level as the criterion) for 72 tests using joint modeling.

The analyses were performed using the jm function from the JMbayes2 [37] in R version 4.0.2. and SAS Enterprise Guide 7.1.

## Results

Of the 6080 participants enrolled for the islet autoantibody follow-up, 5626 children (93%) had food record data available. During the 6-year follow-up, 247 children (4.4%) developed islet autoimmunity at a median (interquartile range [IQR]) age of 2.5 (1.3–3.6) years. Of these 247 children 169 (68%) developed T1D by the age of 15 years. Of the 5674 children (93%) in T1D cohort, 94 children (1.7%) developed T1D during the 6-year follow-up at a median (IQR) age of 4.0 (2.9–5.0) years. Among the 505 children with repeated positivity to at least one autoantibody, the median age at first seroconversion was 1.9 (1.2–3.5) years, and a total of 64 (12.7%) developed T1D by the age of 6 years a median 3.5 (2.4–4.7) years after first seroconversion. The dropout rates among the 5626 participants at 1-, 2-, and 6-year follow-up were 6%, 14%, and 35%, respectively. The total number of food record days from 3 months to 6 years was 81,075 in 5674 children, resulting in average 14.3 food record days per child. Characteristics regarding outcomes by sex, genetic risk, and familial diabetes are presented in Table 1.

**Fig. 1:**
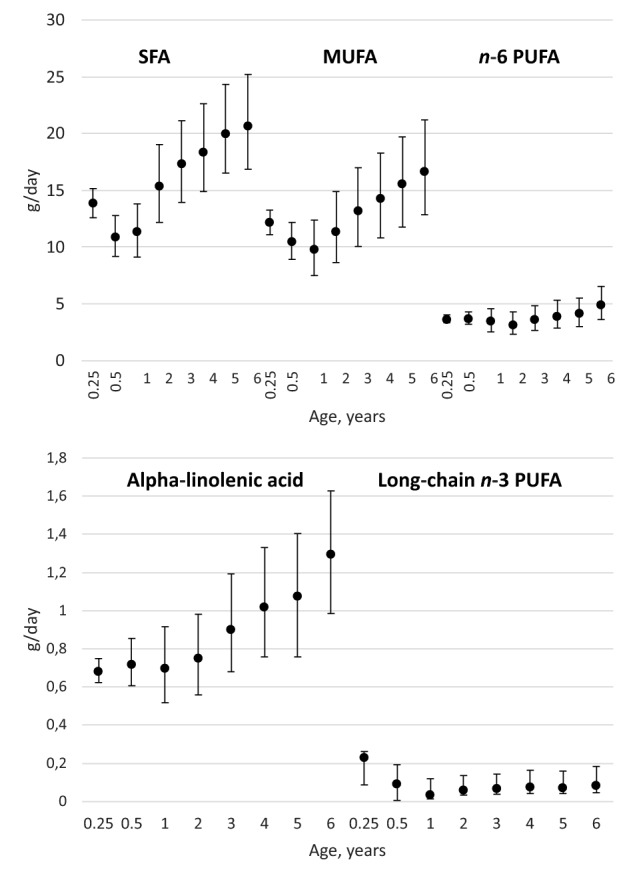
Median (IQR) intake of fatty acids in childhood by age. Number of food record days by age were 15,430 (0.25 years), 14,530 (0.5 years), 13,072 (1 years), 10,752 (2 years), 10,155 (3 years), 9081 (4 years), 1265 (5 years), and 6790 (6 years)

The absolute median intake of most fatty acids showed a J-shaped trend over time such that lowest fatty acid intakes were reported at the age of 1 year (Fig. 1). For long-chain *n*-3 PUFAs the intake was highest in infancy (Fig. 1).

Higher intake of MUFAs, arachidonic acid, *n*-3 PUFAs, and long-chain *n*-3 PUFAs at the age of 3 months to 6 years was associated with a decreased risk of islet autoimmunity with and without energy-adjustment (Table 2), while higher intake of total fat and alpha-linolenic acid was associated with decreased risk of islet autoimmunity only when energy-adjustment was included (Table 2). Higher intake of total fat and SFA, was associated with a decreased risk of T1D in energy-adjusted model but not without energy adjustment (Table 2). Higher intake of SFA, and palmitic acid was associated with a decreased risk of progression from islet autoimmunity to T1D in energy-adjusted model but not without energy adjustment (Table 3).

**Table 2:** Risk of developing islet autoimmunity and type 1 diabetes by children’s fatty acid intake at the age of 3 months to 6 years

N total (*N* outcome)		Islet autoimmunity cohort				Type 1 diabetes cohort			
		5626 (247)				5674 (94)			
	Median (IQR) intake^a^	Adj HR (95% CI)^b,c^	*P*	Energy Adj HR (95% CI)^b,d^	*P*	Adj HR (95% CI)^b,c^	*P*	Energy Adj HR (95% CI)^b,d^	*P*
Total fat, g/day	33.2 (27.1–41.2)	0.98 (0.96, 1.00)	0.137	0.85 (0.75, 0.95)	0.003^f^	1.01 (0.97, 1.04)	0.696	0.73 (0.53, 0.98)	0.032
SFA g/day	13.8 (10.8–17.5)	0.98 (0.94, 1.02)	0.320	0.82 (0.66, 1.01)	0.061	0.99 (0.92, 1.05)	0.735	0.55 (0.33, 0.90)	0.014
Myristic acid (14:0), 100 mg/day	1.7 (1.1–2.2)	0.99 (0.97, 1.02)	0.497	0.92 (0.82, 1.04)	0.180	0.99 (0.95, 1.04)	0.772	0.76 (0.56, 1.00)	0.050
Palmitic acid (16:0), 100 mg/day	7.2 (5.7–8.8)	1.00 (0.99, 1.00)	0.303	0.96 (0.92, 1.00)	0.065	1.00 (0.98, 1.01)	0.610	0.90 (0.81, 1.00)	0.050
MUFA, g/day	11.9 (9.5–14.5)	0.93 (0.88, 0.99)	0.027	0.63 (0.47, 0.82)	< 0.001^f^	1.01 (0.91, 1.11)	0.875	0.53 (0.23, 1.12)	0.104
*n*-6 PUFA, 100 mg/day	36.9 (29.8–45.8)	0.99 (0.97, 1.01)	0.258	0.93 (0.87, 1.00)	0.046	1.01 (0.99, 1.03)	0.370	0.89 (0.73, 1.05)	0.191
Linoleic acid (18:2*n*-6),100 mg/day	35.2 (28.5–44.7)	0.99 (0.97, 1.01)	0.291	0.93 (0.87, 1.00)	0.059	1.01 (0.99, 1.04)	0.296	0.90 (0.75, 1.07)	0.263
Arachidonic acid (20:4*n*-6), 10 mg/day^d^	3.8 (1.5–4.6)	0.89 (0.81, 0.98)	0.018	0.69 (0.50, 0.94)	0.017	1.06 (0.88, 1.25)	0.492	0.95 (0.41, 1.95)	0.936
Conjugated linoleic acid (18:2*n*-6con),10 mg/day	6.2 (4.2–8.1)	1.00 (0.94, 1.05)	0.892	0.90 (0.67, 1.21)	0.505	0.97 (0.86, 1.08)	0.629	0.51 (0.25, 1.03)	0.058
*n-3* PUFA 100 mg/day	10.4 (7.2–15.1)	0.94 (0.89, 0.99)	0.027	0.64 (0.48, 0.84)	0.001^f^	1.03 (0.96, 1.10)	0.377	0.71 (0.37, 1.25)	0.262
Alpha-linolenic acid (18:3*n*-3), 100 mg/day	9.2 (6.3–13.2)	0.95 (0.89, 1.00)	0.075	0.67 (0.49, 0.89)	0.005	1.03 (0.94, 1.11)	0.419	0.72 (0.33, 1.38)	0.369
Long-chain *n*-3 PUFA^e^, 100 mg/day	0.50 (0.24–1.3)	0.44 (0.35, 0.57)	< 0.001^f^	0.14 (0.04, 0.43)	< 0.001^f^	0.83 (0.52, 1.14)^g^	0.493	0.99 (0.31, 2.33)^h^	0.938

^a^Median intakes are based on 81,075 record days across all age points and presented as g/day, 100 mg/day or 10 mg/day. For example, median myristic acids 1.7 100 mg/day corresponds to 170 mg/day

^b^Values are hazard ratios (HR) with 95% credible intervals (CI)s from joint model. Fatty acid intake from 3 months to 6 years and follow-up until 6 years

^c^HR’s are presented per 1 g, 100 mg, or 10 mg increase in nutrient intake. Adjusted for sex, HLA genotype, and familial diabetes

^d^HR’s are presented per gram/MJ, 100 mg/MJ, or 10 mg/MJ increase in nutrient intake. Adjusted for, energy intake, sex, HLA genotype, and familial diabetes

^e^Long chain *n*-3 PUFA includes intake of the following fatty acids: eicosatrienoic acid (20:3 *n*-3), eicosatetraenoic acid (20:4*n*-3), eicosapentaenoic acid (20:5*n*-3), heneicosapentaenoic acid (21:5*n*-3), (22:4*n*-3), docosapentaenoic acid (22:5*n*-3), docosahexaenoic acid (22:6*n*-3)

^f^Statistically significant after multiple testing correction

^g^4th degree polynomial function was used in LME submodel instead of piecewise natural splines due to convergence problems

^h^Three originally chosen knots were replaced by two knots placed at equally long intervals in piecewise natural splines in LME submodel due to convergence problems

**Table 3:** Risk of progression to type 1 diabetes in children with repeated islet autoantibody positivity by fatty acid intake at and after seroconversion

*N* Total (*N* outcome)		Progression cohort			
		505 (64)			
	Median (IQR) intake^a^	Adj HR (95% CI)^b,c^	*P*	Energy Adj HR (95% CI)^b,d^	*P*
Total fat, g/day	39.5 (30.1–51.0)	1.00 (0.96, 1.04)	0.984	0.70 (0.46, 1.08)	0.108
SFA g/day	16.1 (12.0–21.7)	0.98 (0.89, 1.06)	0.580	0.52 (0.27, 0.99)	0.046
Myristic acid (14:0), 100 mg/day	1.7 (1.2–2.4)	0.98 (0.92, 1.03)	0.399	0.67 (0.44, 1.02)	0.063
Palmitic acid (16:0), 100 mg/day	8.0 (6.1–10.4)	0.99 (0.97, 1.01)	0.444	0.86 (0.74, 1.00)	0.046
MUFA, g/day	13.4 (10.2–17.4)	1.01 (0.89, 1.13)	0.884	0.53 (0.18, 1.46)	0.234
*n*-6 PUFA, 100 mg/day	40.3 (30.5–52.4)	1.00 (0.97, 1.03)	0.650	0.93 (0.75, 1.09)	0.446
Linoleic acid (18:2*n*-6),100 mg/day	39.2 (29.4–51.2)	1.00 (0.97, 1.02)	0.757	0.93 (0.76, 1.09)	0.432
Arachidonic acid (20:4*n*-6), 10 mg/day^d^	3.8 (2.1–6.1)	1.15 (0.92, 1.41)	0.233	0.94 (0.16, 4.73)	0.981
Conjugated linoleic acid (18:2*n*-6con),10 mg/day	6.5 (4.4–9.5)	0.95 (0.82, 1.09)	0.499	0.46 (0.16, 1.21)	0.124
*n-3* PUFA 100 mg/day	9.1 (6.8–11.6)	1.05 (0.96, 1.13)	0.240	1.11 (0.56, 2.07)	0.731
Alpha-linolenic acid (18:3*n*-3), 100 mg/day	7.4 (5.9–10.2)	1.07 (0.96, 1.17)	0.189	1.03 (0.51, 1.95)	0.891
Long-chain *n*-3 PUFA^e^, 100 mg/day	0.55 (0.15–2.0)	0.94 (0.49, 1.20)	0.889	1.04 (0.02, 6.36)	0.708

^a^Median intakes are based on 3921 food record days at and after seroconversion presented as g/day, 100 mg/day or 10 mg/day. For example, median myristic acids 1.7 100 mg/day corresponds to 170 mg/day

^b^Values are hazard ratios (HR) with 95% credible intervals (CI)s from joint model. Fatty acid intake from first seroconversion of islet autoantibodies and follow-up until 6 years

^c^HR’s are presented per 1 g, 100 mg, or 10 mg increase in nutrient intake. Adjusted for sex, HLA genotype, familial diabetes, and age at seroconversion

^d^HR’s are presented per gram/MJ, 100 mg/MJ, or 10 mg/MJ increase in nutrient intake. Adjusted for, energy intake, sex, HLA genotype, familial diabetes, and age at seroconversion

^e^Long chain *n*-3 PUFA includes intake of the following fatty acids: eicosatrienoic acid (20:3*n*-3), eicosatetraenoic acid (20:4*n*-3), eicosapentaenoic acid (20:5*n*-3), heneicosapentaenoic acid (21:5*n*-3), (22:4*n*-3), docosapentaenoic acid (22:5*n*-3), docosahexaenoic acid (22:6*n*-3)

Energy intake was associated risk of T1D (HR 1.46; 95% CI 1.10, 1.95 per 1 MJ increase in intake), but not with the risk of IA (1.05; 0.87, 1.25) or progression from islet autoimmunity to T1D (1.35; 0.96, 1.86). When excluding 256 food records 18 months before the T1D diagnosis (*N* = 5660, *N*(T1D) = 80), the energy intake was not associated with the risk of T1D 1.18 (0.73, 1.82), *P* = 0.524). However, the energy-adjusted associations of total fat intake 0.60 (0.36, 0.96), *P* = 0.03 and SFA 0.34 (0.16, 0.73), *P* = 0.003 remained similar to those in the main analyses presented in Table 2.

Intake of fatty acids at 3 to 6 months of age was not associated with the risk of islet autoimmunity or T1D (Online resource, Table 1). We observed no effect modification by breastfeeding at 3 months on the association between early intake of fatty acids and the risk of islet autoimmunity or T1D.

Males had higher intake of SFAs, MUFAs, *n*-6 PUFAs, *n*-3 PUFAs, and total energy than females at 3 to 6 months and at 2 years of age (Online resource, Table 2). Higher maternal education was associated with higher intake of SFAs, MUFAs, and *n*-3 PUFAs and lower intake of *n*-6 PUFAs at 3 to 6 months, and with lower intake of SFA and higher intake of *n*-6 and *n*-3 PUFAs at age of 2 years (Online resource, Table 2). Children with a family member with diabetes had lower intake of SFAs and *n-*3 PUFAs in infancy compared to children without a family member with diabetes, but no such differences were seen at the age of 2. In general, children who were breastfed longer and received higher amount of breastmilk had higher intake of SFAs, MUFAs and *n-*3 PUFAs but lower intake of *n*-6 PUFAs in infancy compared with children with shorter breastfeeding and lower amount of breastmilk (Online resource, Table 3). The following fat variables were associated with total energy intake with Pearson correlation coefficient ≥ 0.60: Total fat (*r* = 0.78), SFA (*r* = 0.72), palmitic acid (16:0) (*r* = 0.67), and MUFA (*r* = 0.68), while other fatty acids showed weaker or no association with energy intake.

## Discussion

Higher intake of several fatty acids in childhood, including intake of *n-*3 fatty acids was associated with decreased risk of developing islet autoimmunity in this study comprising Finnish genetically susceptible children. Higher intake of total fats and SFA was associated with decreased risk of T1D.

The strengths of this study include a large study population and prospective study design, repeated 3-day food record collection, well-maintained food composition database yielding intakes of several individual fatty acids, and long follow-up with regular assessment of islet autoimmunity and T1D. Moreover, joint models allowed for the inclusion of all food records available for each child and, thus, decreasing any bias related to missing data. A limitation is that study is observational, and causality cannot be determined. Further, we could not study interactions with genes related to fatty acid metabolism. This study included children with genetic susceptibility to T1D and it is not known whether the observations can be generalized to all children.

## *N*-3 fatty acids, islet autoimmunity, and T1D

Our findings support the hypothesis that *n*-3 PUFAs and long-chain *n*-3 PUFAs especially could decrease the risk of islet autoimmunity. Our finding that higher total *n*-3 PUFA and alpha-linolenic acid intake is associated with decreased risk of islet autoimmunity is in line with the findings from DAISY study, which found similar associations among all participants (total *n*-3) [12] and among children with increasing number of minor alleles on fatty acid desaturase (FADS1, FADS2) genes (alpha-linolenic acid) [38]. Biomarker studies have reported a direct association [15] or no association [15–18, 38] between alpha-linolenic acid and risk of islet autoimmunity. Our finding that higher long-chain *n*-3 PUFA intake is associated with decreased risk of islet autoimmunity supports the inverse, non-significant association seen in the DAISY study [12]. Biomarker studies have also reported inverse associations between long-chain *n*-3 PUFAs and islet autoimmunity [15–17, 38], however, the association seem to be age, disease endotype and fatty acid specific and such associations are not seen for all long-chain PUFAs, all age points of exposure or at all islet autoimmunity outcomes [15–18, 38]. In this study, we observed a protective association of *n*-3 PUFAs in the models considering intake during the whole age span of 0 to 6 years but not when studying the infancy intakes separately. In DIPP study, we have previously reported associations between serum fatty acids and islet autoimmunity in a nested case–control setting [15, 18]. The associations between fatty acid status and intake with islet autoimmunity differ in some aspects. For example, higher serum alpha-linolenic acid proportion in infancy tended to increase the risk of islet autoimmunity [15], but not at later age points [18]. In the present study energy-adjusted alpha-linolenic acid intake across all ages was associated with decreased risk of islet autoimmunity, but not when intake at infancy was studied separately. The differing results indicate that the no straight-forward conclusions can be made. However, in several studies some of the *n*-3 PUFAs appear important.

We observed protective association of *n*-3 fatty acids with islet autoimmunity but not with T1D. This could indicate that *n*-3 fatty acids play a role in the initiation of the disease process, while other factors may play a stronger role in progression to T1D. Partly different risk factors for islet autoimmunity and development of T1D have been observed in other studies as well [12, 13, 39].

*n*-3 PUFAs and especially, long-chain *n*-3 PUFAs have been suggested to play a role in inflammation, immunity, gut permeability, and gut microbiota [5, 7, 8, 40, 41]. Further, alterations in markers of inflammation, immunity and gut permeability, as well as gut microbiota have been linked with islet autoimmunity or T1D [42–44]. Although there are no studies to support the hypothesis yet, it is possible that higher intake of *n-*3 PUFAs including long-chain *n*-3 PUFAs would decrease the risk of islet autoimmunity in children via one or more of the pathways mentioned. The magnitudes of the associations seem to be clinically meaningful. For example, 100 mg increase in the intake of *n*-3 PUFA is achievable with 1/5 teaspoon of canola oil, and such change was associated with a 6% decrease in the risk of islet autoimmunity.

## Other fatty acids, islet autoimmunity, and T1D

We observed that higher intake of MUFAs and arachidonic acid was associated with a decreased risk of islet autoimmunity with and without energy-adjustment and that higher intake of total fat and *n*-6 PUFA was associated decreased risk of islet autoimmunity only when energy-adjusted. To our knowledge such associations have not been reported before. The DAISY study reported no association between *n*-6 fatty acids and arachidonic acid intake with islet autoimmunity but MUFAs and total fats were not assessed in that study [12]. Biomarker studies report inconsistent observations on the associations between individual MUFAs and *n*-6 PUFAs on the risk of islet autoimmunity [12, 15–17].

Finally, we observed that higher energy-adjusted intake of total fats and SFA was associated with decreased risk of T1D. To our knowledge, these are novel observations and the SFA association is against the initial hypothesis. Hypothetically, higher fat and SFA intake could reflect lower glycemic load of the diet and decrease in postprandial glycemic responses [45]. This could decrease the load of the pancreatic beta cells and delay disease onset [46]. Alternatively, very high fat diet could mimic ketogenic diet [47], which has been suggested to decrease inflammation [48]. However, there are several uncertainties in the causality and mechanisms, and additional studies are therefore needed.

Energy-adjustment affected some of the associations, especially those between fat variables with largest intakes and T1D outcome. The selected energy-adjustments gives us isocaloric interpretation: e.g. What is the risk of T1D when, intake of specific fatty acid increases while energy intake remains the same (and therefore intake from other energy sources: other fatty acids, carbohydrates or proteins, decreases)? Our results indicate that fats, although energy-yielding nutrients, do not increase the risk of T1D and the protective association of total fat and SFA might reflect a risk associated with higher intake of other energy-yielding nutrients. Same phenomenon was seen in progression analyses. Although we observed that reverse causation may explain the association between energy intake and risk of T1D, there was no indication of reverse causality on the protective energy-adjusted associations of total fat and SFA with T1D.

## Conclusions

In this prospective cohort that includes children with an increased risk of T1D, intake of several fatty acids, including intake of *n*-3 fatty acids was associated with a decreased risk of islet autoimmunity. Total fat and SFA intake was associated with the risk of T1D, only when energy was taken into account. Our findings support the idea that dietary factors, including *n*-3 fatty acids, may play a role in the disease process of T1D.

## Supplementary Information

Below is the link to the electronic supplementary material.

Supplementary file1 (DOCX 78 KB)

## Declarations

### Code availability

Data described in the manuscript, and analysis codes can be made available upon request considering the ethical and legislative restrictions.
