# Supplementary material for: Dietary fatty acid intake in childhood and the risk of islet autoimmunity and type 1 diabetes: the DIPP birth cohort study
Source: Eur J Nutr. 2022 Oct 25;62(2):847–56. doi: 10.1007/s00394-022-03035-2 (PMC9941262; doi:10.1007/s00394-022-03035-2)
Supplement: Supplementary file 1 — Supplementary file1 (DOCX 78 KB) [file 394_2022_3035_MOESM1_ESM.docx]

**Dietary fatty acid intake in childhood and the risk of islet autoimmunity and type 1 diabetes: The DIPP birth cohort study**

Authors: Leena Hakola^1,2^, Anna-Leena Vuorinen^1,2,3^, Hanna-Mari Takkinen^1,2,4^, Sari Niinistö^4^, Suvi Ahonen^1,2,4^, Jenna Rautanen^4^, Essi J Peltonen^1,2^, Jaakko Nevalainen^1^, Jorma Ilonen^5^, Jorma Toppari^6,7^, Riitta Veijola^8,9^, Mikael Knip^10,11,12,13^, Suvi M Virtanen^1,2,4, 13^

^1^Faculty of Social Sciences, Unit of Health Sciences, Tampere University, Tampere, Finland

^2^Tampere University Hospital, Research, Development and Innovation Center, Tampere, Finland

^3^VTT Technical Research Centre of Finland, Tampere, Finland

^4^Health and Well-Being Promotion Unit, Finnish Institute for Health and Welfare, Helsinki, Finland

^5^Immunogenetics Laboratory, Institute of Biomedicine, University of Turku, Turku, Finland

^6^Institute of Biomedicine, Research Centre for Integrative Physiology and Pharmacology, and Centre for Population Health Research, University of Turku, Turku, Finland

^7^Turku University Hospital, Department of Pediatrics, Turku, Finland

^8^Department of Pediatrics, PEDEGO Research Unit, Medical Research Center, University of Oulu, Oulu, Finland

^9^Oulu University Hospital, Department of Children and Adolescents, Oulu, Finland

^10^Pediatric Research Center, Children’s Hospital, University of Helsinki and Helsinki University Hospital, Helsinki, Finland

^11^Research Programs Unit, Diabetes and Obesity, University of Helsinki, Helsinki, Finland

^12^Tampere University Hospital, Department of Paediatrics, Tampere, Finland

^13^Center for Child Health Research, Tampere University and Tampere University Hospital, Tampere, Finland

leena.hakola@tuni.fi

**
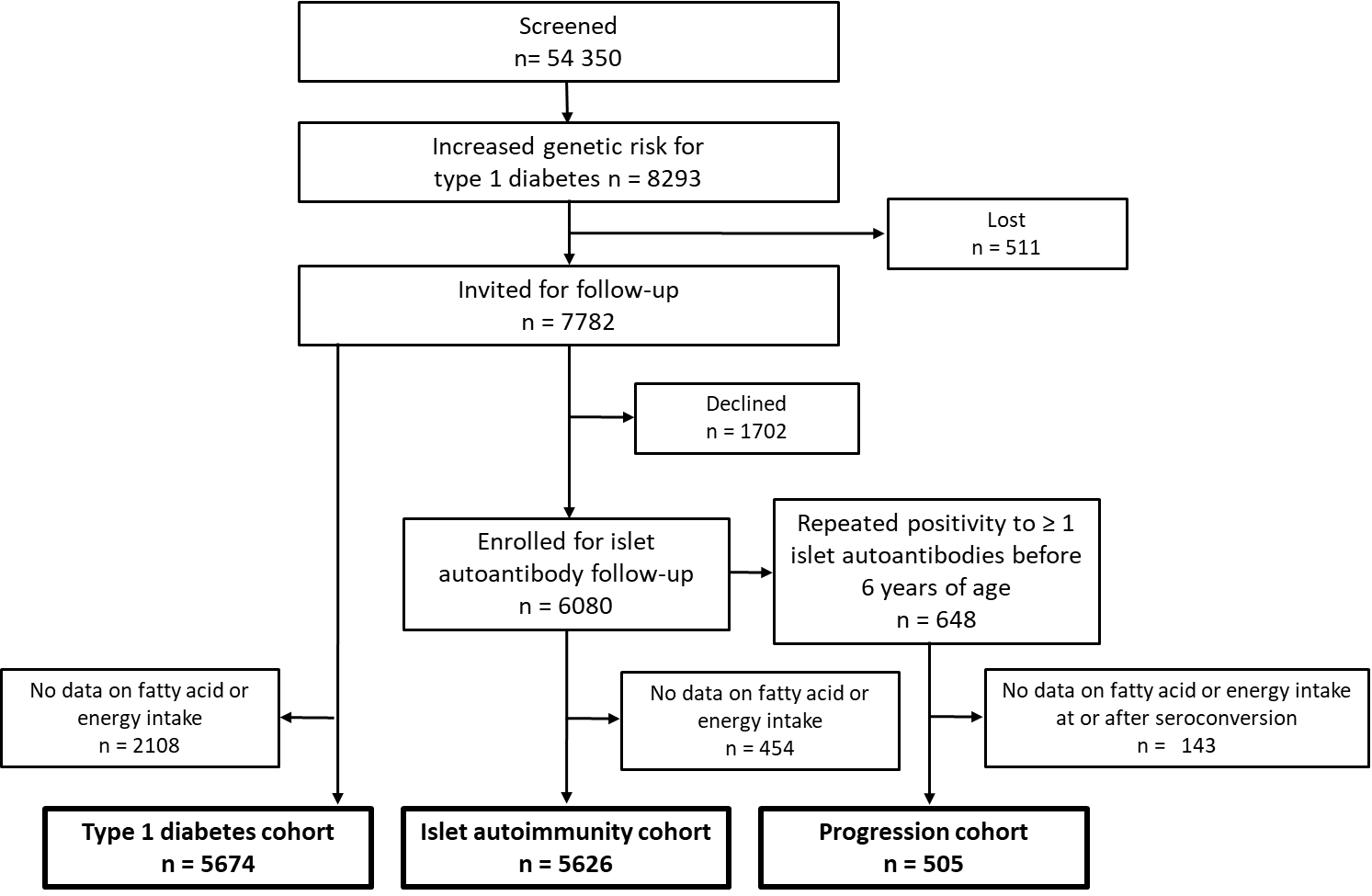
**

**Supplementary Fig. 1.** Study population.

**Supplementary Table 1.** Risk of developing islet autoimmunity and type 1 diabetes by child’s fatty acid intake at the age of 3 months to 6 months.

|  | **Islet autoimmunity** | | | **Type 1 diabetes** | | |
| --- | --- | --- | --- | --- | --- | --- |
|  | **HR** | **(95% CI)^a,b^** | **p-value** | **HR** | **(95% CI)^a,b^** | **p-value** |
| **Total fat, g/MJ day** | 1.04 | (0.92, 1.18) | 0.525 | 1.00 | (0.82, 1.21) | 0.967 |
| **SFA g/MJ day** | 1.06 | (0.84, 1.34) | 0.639 | 0.89 | (0.63, 1.27) | 0.519 |
| Myristic acid (14:0), 100 mg/MJ/day | 0.98 | (0.90, 1.07) | 0.635 | 0.94 | (0.82, 1.07) | 0.335 |
| Palmitic acid (16:0), 100 mg/MJ/day | 1.03 | (0.98, 1.07) | 0.284 | 1.00 | (0.93, 1.07) | 0.934 |
|  |  |  |  |  |  |  |
| **MUFA, g/MJ/day** | 1.12 | (0.82, 1.52) | 0.484 | 1.13 | (0.70, 1.83) | 0.612 |
|  |  |  |  |  |  |  |
| ***n*-6 PUFA, 100 mg/MJ/day** | 1.03 | (0.95, 1.10) | 0.513 | 1.02 | (0.91, 1.15) | 0.691 |
| Linoleic acid (18:2*n*-6),100 mg/day | 1.03 | (0.96, 1.09) | 0.442 | 1.03 | (0.93, 1.14) | 0.594 |
| Arachidonic acid (20:4*n*-6), 10 mg/MJ/day | 0.95 | (0.84, 1.06) | 0.338 | 0.95 | (0.79, 1.14) | 0.578 |
|  |  |  |  |  |  |  |
| **Conjugated linoleic acid** (18:2*n*-6con),10 mg/MJ/day | 0.97 | (0.77, 1.22) | 0.777 | 0.88 | (0.62, 1.24) | 0.457 |
|  |  |  |  |  |  |  |
| ***n-3* PUFA 100 mg/MJ/day** | 0.83 | (0.62, 1.09) | 0.183 | 0.87 | (0.55, 1.36) | 0.531 |
| Alpha-linolenic acid (18:3*n*-3), 100 mg/MJ/day | 0.86 | (0.62, 1.20) | 0.385 | 0.87 | (0.51, 1.47) | 0.596 |
| Long chain *n*-3 PUFA,^c^ 100 mg/MJ/day | 0.89 | (0.61, 1.28) | 0.518 | 0.96 | (0.53, 1.72) | 0.878 |
|  |  |  | |  |  |  |

^a^Values are hazard ratios (HR) with 95% confidence intervals (CI) from Cox proportional hazards regression model. Fatty acid intake was energy-adjusted by dividing the fatty acid intake by energy intake and adding energy as a covariate. Mean energy-adjusted intake of 3 and 6 months was used as an exposure variable. The analyses were further adjusted for sex of the child, HLA genotype and familial diabetes.

^b^HR are presented per 1 grams/MJ 100 mg/MJ, or 10mg/MJ increase in nutrient intake

^c^long chain *n*-3 PUFA includes the following fatty acids: eicosatrienoic acid (20:3 *n*-3), eicosatetraenoic acid (20:4 *n*-3), eicosapentaenoic acid (20:5 *n*-3), heneicosapentaenoic acid (21:5 *n*-3), (22:4 *n*-3), docosapentaenoic acid (22:5 *n*-3), docosahexaenoic acid (22:6 *n*-3)

**Supplementary Table 2.** Mean intake of fatty acids and energy at the age of 3 to 6 months and at 2 years of age by background characteristics.

|  | Mean of intakes at ages 3 and 6 months g/day or kJ/day | | | | |  | Intake at age of 2 years g/day or kJ/day | | | | |
| --- | --- | --- | --- | --- | --- | --- | --- | --- | --- | --- | --- |
|  | SFA | MUFA | *n*-6 PUFA | *n-*3 PUFA | Energy |  | SFA | MUFA | *n*-6 PUFA | *n-*3 PUFA | Energy |
|  | Mean (SD) | Mean (SD) | Mean (SD) | Mean (SD) | Mean (SD) |  | Mean (SD) | Mean (SD) | Mean (SD) | Mean (SD) | Mean (SD) |
| Child sex |  |  |  |  |  |  |  |  |  |  |  |
| Male | 12.8 (2.3) | 11.7 (1.9) | 3.9 (0.7) | 0.9 (0.2) | 2845 (372) |  | 16.3 (5.7) | 12.6 (3.9) | 3.6 (1.4) | 0.99 (0.47) | 4792 (927) |
| Female | 12.0 (2.1) | 11.0 (1.8) | 3.6 (0.6) | 0.8 (0.2) | 2633 (357) |  | 15.5 (5.2) | 11.8 (3.8) | 3.4 (1.4) | 0.92 (0.47) | 4507 (885) |
| *P* value | <0.0001 | <0.0001 | <0.0001 | <0.0001 | <0.0001 |  | <0.0001 | <0.0001 | <0.0001 | <0.0001 | <0.0001 |
| Familial diabetes |  |  |  |  |  |  |  |  |  |  |  |
| Yes | 12.2 (2.1) | 11.2 (1.7) | 3.8 (0.7) | 0.85 (0.16) | 2733 (388) |  | 15.6 (5.6) | 12.0 (3.9) | 3.5 (1.4) | 0.93 (0.41) | 4633 (893) |
| No | 12.5 (2.2) | 11.4 (1.8) | 3.8 (0.7) | 0.88 (0.17) | 2747 (377) |  | 15.9 (5.4) | 12.2 (3.9) | 3.5 (1.4) | 0.96 (0.47) | 4656 (916) |
| *P* value | 0.027 | 0.051 | 0.418 | 0.0012 | 0.740 |  | 0.420 | 0.531 | 0.710 | 0.449 | 0.441 |
| Maternal education |  |  |  |  |  |  |  |  |  |  |  |
| None | 12.2 (2.4) | 11.2 (2.1) | 3.9 (0.8) | 0.85 (0.19) | 2756 (457) |  | 16.6 (5.5) | 12.7 (4.1) | 3.5 (1.5) | 0.91 (0.49) | 4682 (963) |
| Vocational School or Course | 12.3 (2.2) | 11.3 (2.0) | 3.8 (0.8) | 0.86 (0.18) | 2745 (414) |  | 16.6 (5.5) | 12.3 (4.1) | 3.4 (1.4) | 0.88 (0.43) | 4619 (1026) |
| Secondary vocational education | 12.4 (2.2) | 11.3 (1.8) | 3.7 (0.7) | 0.88 (0.17) | 2747 (361) |  | 15.9 (4.9) | 12.2 (3.8) | 3.6 (1.5) | 0.97 (0.48) | 4663 (881) |
| University Studies or Degree | 12.9 (2.2) | 11.6 (1.7) | 3.7 (0.6) | 0.90 (0.15) | 2746 (342) |  | 15.1 (4.9) | 12.0 (3.6) | 3.6 (1.4) | 1.02 (0.47) | 4672 (848) |
| *P* value | <0.0001 | <0.0001 | <0.0001 | <0.0001 | 0.774 |  | <0.0001 | 0.248 | 0.0007 | <0.0001 | 0.327 |

*P* values are from Mann-Whitney or Kruskall Wallis tests.

**Supplementary Table 3**. Intake of fatty acids and energy at the age of 3 to 6 months by breastfeeding status and amount of breastmilk intake.

|  | Mean of intakes at ages 3 and 6 months g/day or kJ/day | | | | |
| --- | --- | --- | --- | --- | --- |
|  | SFA | MUFA | *n*-6 PUFA | *n-*3PUFA | Energy |
|  | Mean (SD) | Mean (SD) | Mean (SD) | Mean (SD) | Mean (SD) |
| Any breastfeeding, months |  |  |  |  |  |
| <3 | 11.5 (2.2) | 10.8 (2.0) | 4.2 (0.79) | 0.78 (0.18) | 2738 (463) |
| 3-5.9 | 12.2 (2.0) | 11.3 (1.8) | 4.0 (0.71) | 0.85 (0.17) | 2794 (399) |
| ≥ 6 | 12.8 (2.2) | 11.5 (1.8) | 3.5 (0.52) | 0.92 (0.15) | 2743 (338) |
| *P* value | <0.0001 | <0.0001 | <0.0001 | <0.0001 | <0.0001 |
| Amount of breastmilk at 3 months |  |  |  |  |  |
| *0* | 11.5 (2.3) | 10.8 (2.1) | 4.19 (0.82) | 0.78 (0.19) | 2717 (465) |
| *low (<median of users)* | 11.7 (1.7) | 10.7 (1.5) | 3.52 (0.66) | 0.83 (0.13) | 2577 (325) |
| *high (≥ median of users)* | 13.8 (1.9) | 12.3 (1.5) | 3.77 (0.44) | 0.97 (0.12) | 2891 (270) |
| *P* value | <0.0001 | <0.0001 | <0.0001 | <0.0001 | <0.0001 |

Median amount of breastmilk among those breastfed at 3 months was 920.16 g/day.

*P* values are from Kruskall Wallis test
